# PD-L1 expression in malignant salivary gland tumors

**DOI:** 10.1186/s12885-018-4069-3

**Published:** 2018-02-06

**Authors:** Koji Harada, Tarannum Ferdous, Yoshiya Ueyama

**Affiliations:** Department of Oral and Maxillofacial Surgery, Yamaguchi University Graduate School of Medicine, 1-1-1 Minamikogushi, Ube, 755-8505 Japan

**Keywords:** PD-L1, Malignant salivary gland tumor, Prognosis, Immunohistochemistry

## Abstract

**Background:**

Programmed death-1 ligand-1 (PD-L1) an important cancer biomarker that can suppress the immune system and its high expression is often reported to be related with increased tumor aggressiveness in some cancers. Here, we examined and evaluated PD-L1 expression in patients with malignant salivary gland tumor. Moreover, the relationship between PD-L1 immunolocalization and clinical pathological features, as well as the prognosis of malignant salivary gland tumors was investigated.

**Methods:**

We examined PD-L1expression in 47 patients with malignant salivary gland tumor by immunohistochemical staining. PD-L1 positivity was defined as ≥5% in tumor cell membrane and evaluated according to three categories (0% = 0, < 5% = 1, ≥5% = 2) in tumor-infiltrating mononuclear cells (TIMCs). Fisher’s exact test was used to compare between PD-L1 expression and clinico-pathological features, and Kaplan–Meier method was used to estimate the distribution of OS by PD-L1 positivity.

**Results:**

PD-L1 expression was detected in 51.1% of malignant salivary gland tumor tissues. No association was observed between PD-L1 immunolocalization in tumor and patient gender, or age. However, PD-L1 immunodetection of tumor cell membranes was significantly associated to stage, recurrence or metastasis after surgery, and patient outcome. On the other hand, PD-L1 immunodetection of tumor-infiltrating mononuclear cells (TIMCs) was significantly associated to recurrence or metastasis after surgery, and patient outcome. PD-L1 positivity in both tumor cell membrane and TIMCs was associated with shorter overall survival (OS) (*p* = 0.002 and *p* = 0.016, respectively).

**Conclusion:**

These findings suggested that patients with PD-L1 positive tumors or TIMCs appear to have poor clinical outcomes in malignant salivary gland tumors.

## Background

The incidence of malignant salivary gland tumors is relatively low compared to other head and neck cancers. They account for more than 0.5% of all malignancies and approximately 3–6.5% of all head and neck cancers [1, 2]. These tumors show varied histological features, and are largely present in the parotid and submandibular glands. The standard treatment for salivary gland cancers is surgical operation because they show resistance to chemotherapy and radiotherapy generally; however, its treatment often requires complex multidisciplinary approach [3, 4]. We often select post-operative radiotherapy when the tumor could not be removed completely by surgery [5]. Unfortunately, these are the only treatment options currently available for malignant salivary gland tumors. As these tumors are often slow growing and are detected at an advanced, non-surgical stage; sometimes they are difficult to treat. Conventional chemotherapies often shows poor efficacy in managing locally advanced or metastatic tumors [3, 4, 6]. Molecular targeted therapies might be useful for the treatment of these patients, although no significant guidelines or tools for selecting candidate patients are available [7]. Thus, novel therapeutic strategies need to be developed and established for the treatment of malignant salivary gland tumors.

It is well known that development and prognosis of malignant tumors are closely associated with host immune functions. Anti-tumor immune responses are induced when the host immune system efficiently identifies the tumor antigen and various T cells are activated. Co-stimulatory molecules and regulative networks play an important role in this progression. There are two groups of co-stimulatory molecules: the tumor necrosis factor TNF receptor (TNFr) superfamily and the immunoglobulin (Ig) superfamily [8]. Programmed death-1 (PD-1) is a co-stimulatory molecule that functions as an immune checkpoint. It is expressed on T cells and pro-B cells, and negatively regulates T cell activation and responses [9]. Two binding ligands have been identified for PD-1, Programmed death-1 ligand-1 (PD-L1, also known as B7-H1) and PD-L2, and both belong to the B7 family [10, 11]. PD-L1 is expressed in resting T cells, B cells, dendritic cells (DCs) and in various tumor cells; and the formation of PD-1 and PD-L1 receptor-ligand complex leads to the inhibition of the cytotoxic T cells and induces special apoptosis of T cells, which results in tumor immune escape [12–14]. Moreover, it has been reported that overexpression of PD-L1 is closely associated with the poor prognosis of renal cell carcinoma, esophageal cancer, gastric cancer, urothelial cancer, pancreatic cancer, and malignant melanoma [12, 15–19]. However, the levels and clinical significance of PD-L1 expression in malignant salivary gland tumor is still unknown.

The purpose of our study was to characterize the PD-L1 expression in patients with malignant salivary gland tumor, and to investigate the relationship between PD-L1 expression levels with clinico-pathological features as well as disease outcomes of the patients.

## Methods

### Patients and samples

Institutional review board (IRB) of the ethical committee of the Yamaguchi University Hospital approved this study (Ref H26–179). Our study was a retrospective one; therefore, informed consent was waived by the IRB.

Forty-seven patients (*n* = 47) with salivary gland cancers (Adenoid cystic carcinoma, Mucoepidermoid carcinoma, Adenocarcinoma and Mucinous adenocarcinoma) treated surgically at Yamaguchi University Hospital from April 1990 to March 2011 were included in this study. Clinico-pathological characteristics such as gender, age, stage and follow-up data (recurrence, metastasis and outcome) were retrospectively collected from patients’ medical records.

### Immunohistochemistry

Tissue samples obtained from biopsy or operation specimens were used to prepare Formalin-Fixed Paraffin-Embedded (FFPE) blocks. Four-micron-thick tumor sections were prepared from these blocks were used for the immunohistochemical analysis. These paraffin-embedded tissue sections were deparaffinized in 100% xylene (Wako Pure Chemical Industries, Ltd.) for 10 min at room temperature, followed by rehydration using graded (100–70% *v*/v, 5 min/each concentration) ethyl alcohol (Muto Pure Chemicals Co., Ltd., Tokyo, Japan). Then, the sections were washed with phosphate-buffered saline (PBS), and heated in a microwave in a Tris-EDTA buffer solution (pH 9.0). The slides were then allowed to cool down, and inserted into a 0.3% hydrogen peroxide/methanol mixture for 20 min at room temperature. After PBS wash, the tissue sections were incubated with Dako REAL™ Peroxidase-Blocking solution (Agilent Technologies, Inc., Santa Clara, CA, USA) for 30 min at room temperature; then incubated overnight at 4 °C with a rabbit polyclonal anti-PD-L1 antibody (Abcam, Cambridge, UK). After PBS wash, Dako REAL™ EnVision™ Detection system (Agilent Technologies) was used according to the manufacturer’s protocol to detect the immunostaining. Tissues were then lightly counterstained with hematoxylin (Muto Pure Chemicals Co., Tokyo, Japan), and were subsequently dehydrated in graded (70–100% *v*/v) ethyl alcohol (Muto Pure Chemicals Co., Ltd.), inserted in xylene (Wako Pure Chemical Industries Ltd.) and mounted with glass coverslips using DPX mounting medium (Sigma-Aldrich; Merck KGaA). In case of negative controls, primary antibody was omitted.

### Quantification of PD-L1 expression in tumor cell membrane

**Fig. 1:**
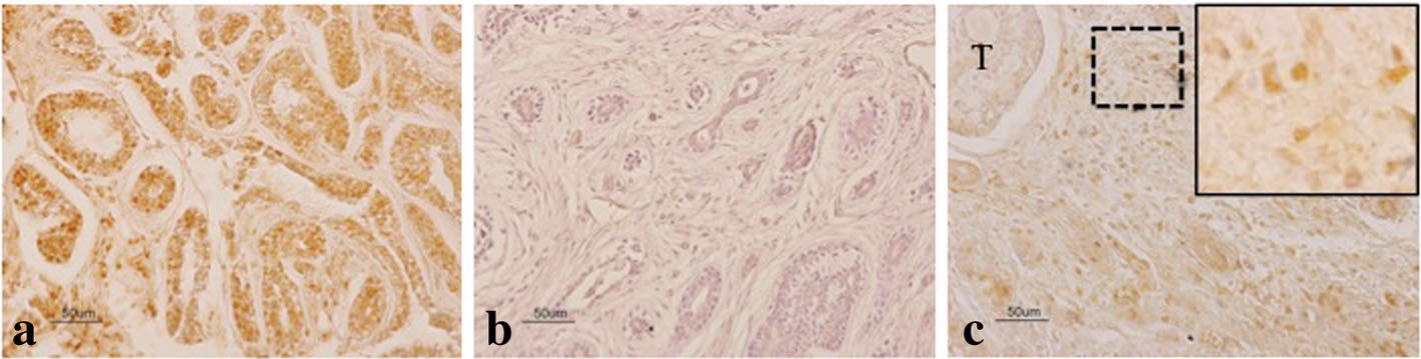
PD-L1 expression in FFPE samples stained with anti-PD-L1 antibody. **a** Positive staining is present in tumor cells membrane. **b** Negative staining is present in tumor cells membrane. **c** Tumor cells are negative (T) and TIMCs are positive for PD-L1, magnified view of TIMCs was shown in the upper right corner

Immunoreactivity for PD-L1 expression was evaluated in tumor cell membrane by three authors (KH, TF, and YU), who had no knowledge of the patient’s clinical status. Briefly, five randomly selected areas were examined. The proportion of tumor cells showing high and low immunolabeling in each selected field was determined by counting individual tumor cells at high magnification (× 400). At least 200 tumor cells were counted. PD-L1 tumor positivity was defined as ≥5% tumor cell membrane staining. PD-L1-positive immunolabeling was predominantly located in the cytoplasm and with some nuclear membrane localization (Fig. 1).

### Quantification of PD-L1 expression in tumor-infiltrating mononuclear cells

The extent of tumor-infiltrating mononuclear cells (TIMCs) (i.e. lymphocytes and macrophages) was assessed in hematoxylin and eosin-stained slides and evaluated as absent (0), focal (1), mild (2), moderate (3) and marked (4) by three authors (KH, TF, and YU), who had no knowledge of the patient’s clinical status. The percentage of PD-L1-positive TIMCs was also evaluated independently. Immunoreactivity for PD-L1 expression was evaluated according to three categories (0% = 0, < 5% = 1, ≥5% = 2). An adjusted score representing PD-L1 expression was calculated multiplying the percentage of TIMCs that stained positive for PD-L1 and the extent of mononuclear cell infiltration.

### Statistical analysis

In the present study, we investigated PD-L1 expression and its association with clinical outcome in patients with malignant salivary gland tumor. Overall survival (OS) defined as time from diagnosis to death was analyzed as an end point. In the absence of an event, the end point was censored at last follow-up time. Patient and tumor characteristics were summarized descriptively. PD-L1 positivity was defined as 5% or greater of tumor cell membrane staining. For PD-L1 expression in TIMCs, any score greater than five was considered high. Comparisons between PD-L1 expression and clinico-pathological features were evaluated using Fisher’s exact test for categorical variables. Kaplan–Meier method estimated the distribution of OS by the PD-L1 positivity. All statistical computations were carried out using the StatView software (version 5.0 J, SAS Institute Inc. Cary, NC, USA) and a *p* value (two-sided) < 0.055 was considered statistically significant.

## Results

### Patients and tumor characteristics

Characteristics of 47 patients with malignant salivary gland tumor included in this study are summarized in Table 1. The histological subtypes included adenoid cystic carcinoma (*n* = 25), mucoepidermoid carcinoma (*n* = 9), adenocarcinoma (*n* = 11) and mucinous adenocarcinoma (*n* = 2). The median follow-up time was 7.4 years [interquartile range (IQR): 1.8–12.8], and the median age was 62 years (range 24–80 years). For malignant salivary gland tumor, clinical stages I, II, III and IV at diagnosis was identified in 9, 18, 5 and 15 patients, respectively.

**Table 1:** Patient characteristics

Characteristic	Total (*n* = 47) No. of patients	%
Gender		
Male	26	55.3
Female	21	44.7
Stage		
I	9	19.1
II	18	38.3
III	5	10.6
IV	15	31.9
Histology		
adenoid cystic carcinoma	25	40.4
mucoepidermoid carcinoma	9	17.0
adenocarcinoma	11	23.4
mucinous adenocarcinoma	2	6.4
Recurrence or metastasis after surgery		
No	31	66.0
Yes	16	34.0
PD-L1 expression in tumor cells membrane		
< 5% (negative)	23	48.9
≥ 5% (positive)	24	51.1
PD-L1 expression in tumor-infiltrating mononuclear cells (TIMC)		
Score < 4 (negative)	27	57.4
Score ≥ 4 (positive)	20	42.6
	Median	Min-max
Age (years)	62.0	24–80

### PD-L1 expression in tumor cells and clinico-pathological features

Table 2 shows the association between PDL-1 expression in tumor cell membrane and clinico-pathological features of patients. Among 47 patients with malignant salivary gland tumor, 23 patients (48.9%) showed negative PD-L1 expression in tumor cell membrane; whereas 24 patients (51.1%) showed positive expression. Specifically, PD-L1 positivity in tumor cell membrane was detected in 11 of 25 (44.0%) adenoid cystic carcinoma patients, 7 of 11 (63.6%) mucoepidermoid carcinoma patients, 5 of 9 (55.6%) adenocarcinoma patients, and 1 of 2 (50.0%) mucinous adenocarcinoma patients. PD-L1 positivity in tumor cell membrane was significantly associated with higher stage (*p* = 0.047), recurrence or metastasis after surgery (*p* = 0.028), and fatal outcome (*p* = 0.002). On the other hand, PD-L1 positivity was not associated with gender, age at diagnosis.

**Table 2:** Association between PD-L1 expression in malignant salivary gland tumor membrane and clinico-pathological factors^a^

Characteristic	% Positive tumor cell membrane			*p*-value
	< 5% (negative) (*n* = 23, 48.9%), *n*	5% or more (positive) (*n* = 24, 51.1%), *n*	Total (*n* = 47)‚ *n*	
Gender				> 0.999
Male	13	13	26	
Female	10	21	21	
Age				> 0.999
65≥	13	12	25	
65<	10	12	22	
Stage				0.047
I + II	17	10	27	
III + IV	6	14	20	
Recurrence or metastasis after surgery				0.028
No	19	12	31	
Yes	4	12	16	
Outcome				0.002
Alive	22	13	35	
Death	1	11	12	

^a^Fisher’s exact test

### PD-L1 expression in TIMCs and clinico-pathological features

Association between PDL-1 expression in TIMC and clinico-pathological factors are presented in Table 3. Overall, the extent of TIMCs infiltration was: absent in 0 patients, focal in 23 patients, mild in 17 patients, moderate in 6 patients and marked in 1 patient. PD-L1 expression in TIMCs was low (score < 5) in 27 patients (57.4%). Twenty patients (42.6%) were considered as PD-L1 high (score ≥ 5) in the TIMCs. Among the cases with PD-L1 high TIMCs, all patients showed positive expression in more than 5% of immune cells. There was a significant association between PD-L1 expression levels in TIMCs and recurrence or metastasis after surgery (*p* = 0.011), as well as PD-L1 expression levels and outcome of patients (*p* = 0.049). PD-L1 positivity in TIMCs was not significantly associated with gender (*p* > 0.999), age (*p* = 0.769), stage (*p* = 0.073).

**Table 3:** Association between PD-L1 expression in tumor-infiltrating mononuclear cells (TIMCs) and clinico-pathological factors^a^

Characteristic	% Positive tumor- infiltrating mononuclear cells			*p*-value
	Score < 5 (low) (*n* = 27, 57.4%), *n*	Score ≥ 5 (high) (*n* = 20, 42.6%), *n*	Total (*n* = 47)‚ *n*	
Gender				> 0.999
Male	15	11	26	
Female	12	9	21	
Age				0.769
65≥	14	11	25	
65<	13	9	22	
Stage				0.073
I + II	19	8	27	
III + IV	8	12	20	
Recurrence or metastasis after surgery				0.011
No	19	12	31	
Yes	8	8	16	
Outcome				0.049
Alive	23	12	35	
Death	4	8	12	

^a^Fisher’s exact test

### PD-L1 expression in malignant salivary gland tumors and survival time

**Fig. 2:**
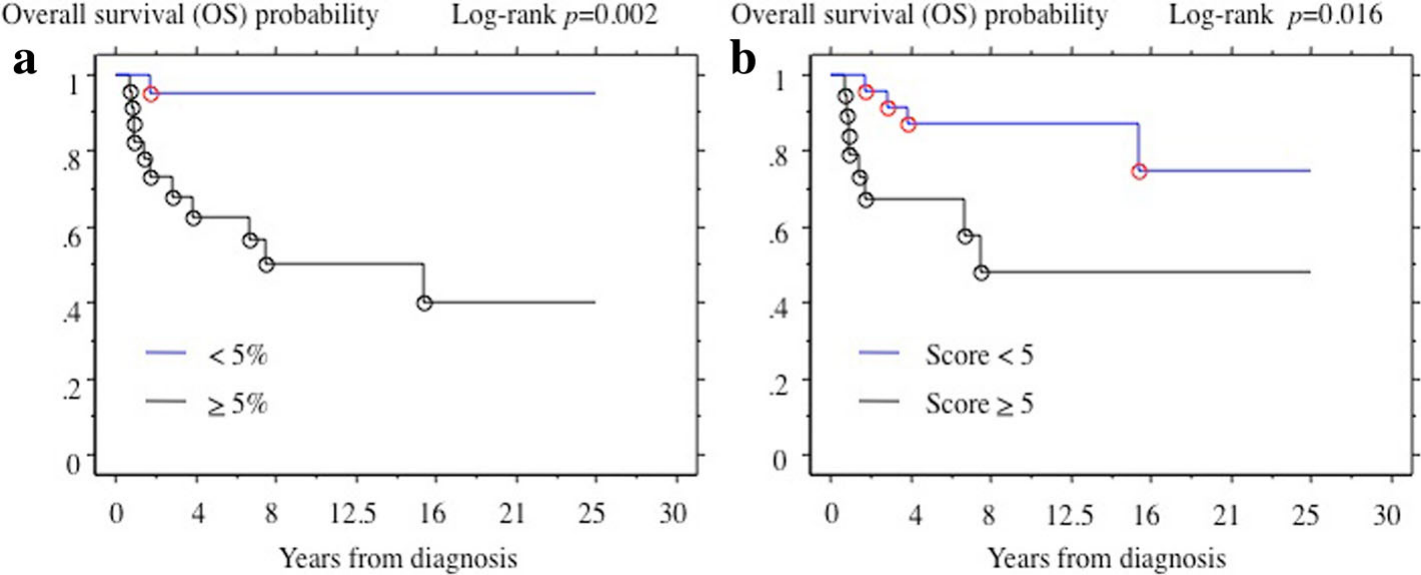
PD-L1 expression in malignant salivary gland tumors. **a** Association of PD-L1 expression and OS in malignant salivary gland tumors. **b** Association of PD-L1 expression and OS in TIMCs. OS by PD-L1 positivity was estimated by Kaplan–Meier method. **a** PD-L1 positivity on tumor membrane (% positive neoplastic cell). **b** PD-L1 Expression in TIMC (inflammatory cell store)

The overall median follow-up of the cohort was 7.4 years, 12 patients died and 16 patients had recurrence or metastasis after surgery. PD-L1 positivity on tumor cell membrane and TIMCs both were associated with OS (*p* = 0.002 and *p* = 0.016, respectively) (Fig. 2).

## Discussion

This study demonstrated significant association between PD-L1 positivity in tumor cells and higher stage, recurrence or metastasis after surgery, fatal outcome or survival time in patients with malignant salivary gland tumors. In addition, significant association was also observed between PD-L1 expression levels in TIMCs and recurrence or metastasis after surgery or patient outcome. Therefore, we can assume that patients with PD-L1 positive malignant salivary gland tumors or TIMCs cannot be expected to show favorable prognosis, especially when conventional therapy or surgical operation is used as the treatment method. Hence, novel and advanced therapeutic approaches against locally advanced or metastatic malignant salivary gland tumors are needed to ensure favorable treatment outcome of patients. It was reported that, blocking the interactions between PD-1 and PD-L1 can inhibit antitumor immunity, enhance T-cell-mediated immune function and promote antitumor activity of therapeutic agents in preclinical models and in vitro [20]. Brahmer et al. showed the efficacy of anti-PD-L1 antibody (BMS-93655) in his multicenter phase 1 trial study with patients with advanced cancer, including non-small-cell lung cancer, melanoma and renal-cell cancer [20]. Recently, anti-PD-1 monoclonal antibody (nivolumab) is considered as a new therapeutic option for the treatment of unresectable malignant melanoma [21]. Moreover, some clinical studies that evaluated the safety and efficacy of nivolumab in patients with advanced cancer showed encouraging results [21–24]. However, higher PD-L1 expression in tumor cells is not always associated with unfavorable outcome [25, 26].

Until now, there were no available reports that indicated any relationship between PD-L1 expression and clinical outcomes in malignant salivary gland tumor patients. To our knowledge, this is the first study that demonstrated the association between PD-L1 expression in tumor cells or TIMCs with recurrence or metastasis after surgery, fatal outcome and shorter OS in patients with malignant salivary gland tumors treated by surgical operation. We could detect PD-L1 expression both in tumor cells and TIMCs. The detail mechanisms of PD-L1 expression in tumor cells or TIMCs are still unknown. It is reported that, Natural killer cells as well as T-cells expresses cytokines such as interferon-γ (IFN-γ), tumor necrosis factor-α (TNF-α) and interleukin-2 (IL-2) which in turn can induce PD-L1 expression on surrounding immune and tumor cells when T-cells recognize antigen and become activated [27]. TIMCs also have an important role in induction of PD-L1 expressions, and various cytokines derived from TIMCs promotes tumor growth as well as impairs antitumor immune responses. The expression mechanisms of PD-L1 must be complicated with different circumstances involved; however, the extent of TIMCs could be an important predictive factor for anti-PD-1 monoclonal antibody therapy including nivolumab [21]. In this study, we found high extent of TIMCs, as well as high PD-L1 expression in both tumor cells and TIMCs. Nivolumab has just got marketing approval as a drug for the treatment of unresectable malignant melanoma, and it might also be effective for locally advanced or metastatic malignant salivary gland tumors [21]. Further in vitro, in vivo and clinical studies with anti-PDL-1 antibody (BMS-93655) against salivary gland tumors might also generate favorable results.

Our study is a retrospective analysis of PD-L1 expression and we have only analyzed 47 cases of malignant salivary gland tumors. Further prospective studies are needed to understand the role of PD-L1 expression more precisely in immune cells as a predictive and prognostic biomarker in malignant salivary gland tumors.

## Conclusion

This is the first study that demonstrated the association between PD-L1 positive expression and clinical stage, recurrence or metastasis after surgery and survival time of patients with malignant salivary gland tumors. Our data also showed significant association between PD-L1 expression levels in TIMCs and recurrence or metastasis after surgery or patient outcome.

## Abbreviations

DC: dendritic cells
FFPE: Formalin-Fixed Paraffin-Embedded
IFN-γ: Interferon-γ
Ig: Immunoglobulin
IL-2: Interleukin-2
OS: Overall survival
PBS: Phosphate-buffered saline
PD-1: Programmed death-1
PDL1: Programmed death ligand 1
TIMC: Tumor-infiltrating mononuclear cell
TNFr: TNF receptor
TNF-α: Tumor necrosis factor-α
